# Supplementary material for: Mothers’ health care seeking behavior for neonatal danger sign in southern Ethiopia: Community based cross–sectional study
Source: PLoS One. 2023 Jul 19;18(7):e0280993. doi: 10.1371/journal.pone.0280993 (PMC10355418; doi:10.1371/journal.pone.0280993)
Supplement: S1 File — (DOCX) [file pone.0280993.s001.docx]

## 10.2 Annex II Questionnaire English Version

**Instruction: -** Circle the responses for questions with alternatives and write for open ended questions on the space provided.

**Part I: Socio demographic characteristics**

| **S.No** | **Questions** | **Responses** |
| --- | --- | --- |
|  | How old are you? | 1. 15-20 yrs old 2. 21-30 yrs old 3. 31-40yrs old |
|  | Marital status | 1. Single 2. Married 3. Divorced 4. Widowed |
|  | What is your educational status | 1. Never Attending School 2. Grade 1-6^th^ 3. Grade 7-12^th^ 4. College 5. University 6. Other (specify)_____ |
|  | What is your Husband’s educational status? | 1. Never Attending School 2. Grade 1-6th 3. Grade 7-12th 4. College 5. University 6. Other (specify)_____ |
|  | What is your occupational status | 1. Merchant 2. Government Employee 3. Housewife 4. Daily worker 5. Student 6. Other (specify) _____ |
| 8. | What is your husband’s Occupation? | 1. Merchant 2. Government Employee 3. Daily worker 4. Student 5. Farmer |
| 9. | What is your estimated monthly income? | 1. I have not 2. 500-1000ETB 3. 1500- 3000 ETB 4. >35000 ETB |
| 10. | What type of communication media used | 1. Television 2. Radio |
| 11. | Where is your residence? | 1. Urban 2. Rural |

**Part II. Obstetric information**

Instruction: circle or write the responses for questions with alternative answer/s.

| **S.No** | **Questions** | **Responses** |
| --- | --- | --- |
|  | How many times have you been pregnant? | 1. One time 2. Two times 3. Three & above |
|  | How many times did you give birth? | 1. One time  2. Two times  3. Three & above |
|  | \|  \|  \| \| --- \| --- \|   How many alive children do you have? | 1. No 2. One 3. Two 4. Three 5. Above three |
|  | Have you ever had an ANC follow up? | 1. Yes 2. No |
|  | If yes, how many ANC follow up did you had? | 1. <4  2. ≥4 |
|  | Did you have PNC follow up? | 1.Yes  2. No |
|  | If yes, number of PNC visits | 1. <3  2. ≥3 |
| 8. | Where did you deliver your current baby? | 1. Home 2. Health center 3. Hospital 4. Other(specify)---------- |
| 9 | How did you deliver? | 1. Spontaneous vertex delivery 2. Cesarean section 3. Instrumental delivery |

**Part III Practice of mothers on neonatal danger sign**

| **S.no** | **Question** | **Response** |
| --- | --- | --- |
|  | Danger signs that new-born experience | 1. Persistent vomiting 2. Convulsion 3. Breathing problem 4. Fever 5. Diarrhea 6. Jaundice |
| **1** | Have you ever seen a sick neonate in your own family? | 1. Yes 2. No |
| **2** | If your newborn has any of  Manifestations of illness what did you do? | 1. Take to Health institution 2. I gave Home treatment 3. Take to Traditional healer 4. Do nothing |
| **3** | Where did you seek medical care for your sick neonates? | 1. Government health institution 2. Private clinic 3. Pharmacy 4. Other (specify) |
| **4** | If a neonate have diarrhea what will you do? | 1. Take to traditional healer 2. I give home treatment 3. Take to health institution 4. Do nothing |
| **5** | If a neonate have persistent  Vomiting what will you do? | 1. Stop breast feeding 2. I give home treatment 3. Take to health institution 4. Take to traditional healer 5. Do nothing |
| **6** | What will you did if the neonate convulse? | 1. Take to health institution 2. I gave Home treatment 3. Take to Traditional healer 4. Do nothing |
| **7** | What will you did if the neonate face breathing problem? | 1. Take to health institution 2. Take to Traditional healer 3. Do nothing |
| **8** | What will you did if the neonate becomes febrile? | 1. Take to health institution 2. I gave Home treatment 3. Take to Traditional healer |
| **9** | What will you did if the neonate faced by jaundice? | 1. Take to health institution 2. I gave Home treatment 3. Take to Traditional healer 4. Do nothing |
| **10** | Reasons for not seeking medical care | High treatment cost  Illness was not serious  Considering that home remedies are more effective  Didn’t trust facility/poor quality of care  Lack of knowledge about danger signs  No reason |

**Thank you for your participation**
