# Supplementary material for: Mothers’ health care seeking behavior for neonatal danger sign in southern Ethiopia: Community based cross–sectional study
Source: PLoS One. 2023 Jul 19;18(7):e0280993. doi: 10.1371/journal.pone.0280993 (PMC10355418; doi:10.1371/journal.pone.0280993)
Supplement: Attachment — Submitted filename: Response lette.docx [file pone.0280993.s003.docx]

**Response letter**

Dear editors and reviewers:

Thank you for considering my manuscript for publication in your reputable journal. I have noted that your comments are valuable and make my research article well improved. I have made appropriate changes in the main document. Here in below are the point by point responses for your concern.

**Academic Editor Comments to the Author**

1. Please ensure that your manuscript meets PLOS ONE's style requirements, including those for file naming.

- **Author’s response:** the manuscript revised and written with PLOS ONE's style

2. Please amend your current ethics statement to address the following concerns:

a) Did participants provide their written or verbal informed consent to participate in this study?

b) If consent was verbal, please explain i) why written consent was not obtained, ii) how you documented participant consent, and iii) whether the ethics committees/IRB approved this consent procedure.

- **Author’s response:** participants get verbal informed consent to participate in this study. I have no special reason to give verbal informed consent to participants. It is well explained on ethical approval and consent to participate section. During data collection respondents were requested their willingness to participate; those who were willing to participate were included in this study, but those who were not willing to participate were excluded from the study. Ethical approval was obtained from Wolaita Sodo University College of health science and medicine Ethical review board

3. You indicated that you had ethical approval for your study. In your Methods section, please ensure you have also stated whether you obtained consent from parents or guardians of the minors included in the study or whether the research ethics committee or IRB specifically waived the need for their consent.

- **Author’s response:** Oral informed consent was obtained from the parents of the minors

4. Please include additional information regarding the survey or questionnaire used in the study and ensure that you have provided sufficient details that others could replicate the analyses. For instance, if you developed a questionnaire as part of this study and it is not under a copyright more restrictive than CC-BY, please include a copy, in both the original language and English, as Supporting Information.

- **Author’s response:** I will include a copy of questionnaire as Supporting Information.

5. . Thank you for stating the following financial disclosure:

“This study did not obtain any particular funding from govern.ent, commercial, or non-profit funding agencies.”

At this time, please address the following queries:

a) Please clarify the sources of funding (financial or material support) for your study. List the grants or organizations that supported your study, including funding received from your institution.

b) State what role the funders took in the study. If the funders had no role in your study, please state: “The funders had no role in study design, data collection and analysis, decision to publish, or preparation of the manuscript.”

c) If any authors received a salary from any of your funders, please state which authors and which funders.

d) If you did not receive any funding for this study, please state: “The authors received no specific funding for this work.

Please include your amended statements within your cover letter; we will change the online submission form on your behalf.

- **Author’s response:** This study did not obtain any particular funding from government, commercial, or non-profit funding agencies

6. Thank you for stating the following in your Competing Interests section:

“There is no competing interest”

Please complete your Competing Interests on the online submission form to state any Competing Interests. If you have no competing interests, please state ""The authors have declared that no competing interests exist."", as detailed online in our guide for authors at <http://journals.plos.org/plosone/s/submit-now>

 This information should be included in your cover letter; we will change the online submission form on your behalf.

- **Author’s response:** I will complete your Competing Interests on the online submission

**Reviewer Comments to the Author**

1. edit title to read maternal health seeking behavior

- **Author’s response:** the title edited as health seeking behavior

2. focus your literature on WHO danger signs. Which danger signs were studied and why? specify and justify.Wat guidelines are used in Ethiopia for neonatal care? how does a mother learn about these signs, if they don't attend anc/pnc? are there community health workers in Ethiopia?

- **Author’s response:** Convulsions, fever, lethargy, and poor breast milk feeding, as well as chest retractions, jaundice, and vomiting are neonatal danger signs this study focus. Most of the time children death because of the above listed neonatal danger signs. INTEGRATED MANAGEMENT OF NEWBORN AND CHILDHOOD ILLNESS and guidelines are used in Ethiopia for neonatal care. Health extension workers play a great role in Ethiopia to teach mothers about the neonatal danger signs and mothers who have ANC/PNC follow up get information from health care providers.

3. METHODS: is it a retrospective study? how do you deal with recall bias? indicate ethical approval number.

- **Author’s response:** data was collected from mothers who give birth in the last 12 months preceding the survey so I have not face with recall bias. Mothers mostly didn’t forget what happened on their children. ethical approval reference number is CHSM/ERC/08

4. RESULTS: ensure all percentages add to 100%, seen many errors.summarize the tables- combine column 2 and 3 eg. 169(41.2%). quantify herbal use meds. how is husband s education important in this study?

- **Author’s response:** all percentages were added to 100%. In Ethiopia husbands have a great influence on all aspects of their wife.

5. Discuss the results and don't repeat results here. requires to be re written focusing on neonate, danger signs and mother. which danger sign was highly missed and why? this will help to implement better guidelines and health seeking behavior.

- **Author’s response:** the discussion part have revised

6. An assessment of the knowledge, and the attitudes in addition to the practice would have added a significant lot of value to validate the results. And to assess the practice, the mothers actually needed to have a knowledge of the danger of these danger signs. The factors associated with health care seeking practice identified in the study such as husband’s education status, access to media, urban residence, ANC follow ups etc, clearly point towards access ‘Knowledge’.
   The writing could also be improved with the help of a native English editor.

- **Author’s response:** prior to assess the practice or behavior the knowledge and the practice part have assessed separately. The manuscript had revised with native English editor.

7. Online 34, the authors need to indicate what statistical tests were carried out before the sentence “A p-value of less than or equal to 0.05 in a multivariable……..”

- **Author’s response:** on abstract**,** before writing about p-value the statistical test indicated.

8. On pg 10, line 91, the sentence that starts with “Solitary limited studies remained conducted in Ethiopia with respect to mothers’ practice towards neonatal danger signs” should be referenced. The findings in this solitary study should be mentioned and other studies in the region could be reviewed as well. The gaps in those other studies should then be outlined that this particular study promises to close. The real scientific addition from the study should then be articulated after this sentence: “ So this research is intended to assess the ……” lines 92-94

- **Author’s response:** the term used ‘Solitary’ is not to stat only but to explain as little or no enough study have conducted in Ethiopia. Please understand what I want to say.

9. If the authors insist on only assessing the practice of health seeking for danger signs, then the study population should only include those mothers who recognized danger signs in their babies, and the sample size calculated should only be for these population. There is no need to include the mothers whose babies had no danger signs in the initial interviews and in the analysis.
   The sampling procedure on Pg 11, lines 116-19 is not clear, how the coding was done and every 6th woman is selected- these need to be explained in more detail. As already stated above, the effective sample size in this study so far is 231 NOT 410. If it is logistically difficult to get more participants into the study, perhaps the authors should acknowledge this as a limitation in the discussion sections.

- **Author’s response:** even if the authors intention is to see health care practice of mother for neonatal danger sign, from 410 we get 231(56.3%) of mothers had encountered at least one of the WHO-recognized neonatal danger signs. So we observe the prevalence of neonatal danger sign which is 56.3% prior talking about practice of mothers who face neonatal danger sign.
