# Supplementary material for: Mothers’ health care seeking behavior for neonatal danger sign in southern Ethiopia: Community based cross–sectional study
Source: PLoS One. 2023 Jul 19;18(7):e0280993. doi: 10.1371/journal.pone.0280993 (PMC10355418; doi:10.1371/journal.pone.0280993)
Supplement: Attachment — Submitted filename: Response lette.docx [file pone.0280993.s005.docx]

**Response letter**

Dear editors and reviewers:

Thank you for considering my manuscript for publication in your reputable journal. I have noted that your comments are valuable and make my research article well improved. I have made appropriate changes in the main document. Here in below are the point by point responses for your concern.

**Reviewer Comments to the Author**

1. The first sentence line 54 requires an addition of a reference. The sentence is also technically confusing. Which is the newborn period? As far as I know, this period is up to one month while the infants are up to one year.

- **Author’s response:** The sentence and the reference have corrected. As WHO define, ‘A newborn infant, or neonate, is a child under 28 days of age’. The reviewers saying as it is written in revised version.

2. The authors acknowledge on line 77 pg13 that the ‘knowledge’ of danger signs influence the ‘practice’ of health seeking and their response is simply that this has been assessed separately. I suggest the authors include this in their literature review under introduction and provide the knowledge levels that currently exist

- **Author’s response:** the current knowledge level have been incorporated under introduction from a systematic review conducted in Ethiopia

3. The response “the term used ‘Solitary’ is not to stat only but to explain as little or no enough study have conducted in Ethiopia. Please understand what I want to say” is still not adequate in my view. I see the word ‘solitary’ has been changed to ‘limited’ but the issue raised in the comment was actually to discuss the findings of these limited studies and identify the gaps that this study would like to address. This is usually the origin of new studies covering the same subject.
   **Author’s response:** dear reviewer I understand what you want to say. Some research has done in Ethiopia but it is not enough regarding this very critical area because most women loss their children due to neonatal danger sign.
4. In methods section: The issue of sampling and sample size remains largely unaddressed. Yes, the prevalence of danger signs is 56.3% but this is not what the study is set up for. It is the those who developed these danger signs we are interested in- how many sought care and what influenced their behavior?

- **Author’s response:** numbers of mothers seeking care from health institution and factors influenced their behavior have stated in the manuscript
